# Chemical Composition, Antioxidant, and Antibiofilm Properties of Essential Oil from *Thymus capitatus* Plants Organically Cultured on the Greek Island of Lemnos

**DOI:** 10.3390/molecules28031154

**Published:** 2023-01-24

**Authors:** Eirini Maniki, Dimitra Kostoglou, Nikolaos Paterakis, Anastasios Nikolaou, Yiannis Kourkoutas, Alexandros Papachristoforou, Efstathios Giaouris

**Affiliations:** 1 Laboratory of Food Microbiology and Hygiene, Department of Food Science and Nutrition, School of the Environment, University of the Aegean, 81400 Myrina, Lemnos, Greece; 2 Aegean Organics, Organic Herbs and Essential Oils, 81400 Agios Dimitrios, Lemnos, Greece; 3 Laboratory of Applied Microbiology and Biotechnology, Department of Molecular Biology and Genetics, Democritus University of Thrace, 68100 Alexandroupolis, Evros, Greece; 4 Department of Food Science and Technology, School of Agriculture, Aristotle University of Thessaloniki, 57001 Thermi, Thessaloniki, Greece

**Keywords:** thyme essential oil, gas chromatography, antioxidant activity, total phenolic content, foodborne bacterial pathogens, antibacterial action, antibiofilm action, food safety, health promotion

## Abstract

Essential oils (EOs) are mixtures of volatile plant secondary metabolites and have been exploited by humans for thousands of years for various purposes because of their many bioactivities. In this study, the EO from *Thymus capitatus*, a thyme species organically cultured on the Greek Island of Lemnos, was analyzed for its chemical composition (through GC-FID and GC-MS), antioxidant activity (AA), and total phenolic content (TPC), as well as its antimicrobial and antibiofilm actions against three important foodborne bacterial pathogens (*Salmonella enterica* ser. Typhimurium, *Listeria monocytogenes*, and *Yersinia enterocolitica*). For the latter investigations, the minimum inhibitory concentrations (MICs) and minimum biofilm inhibitory concentrations (MBICs) of the EO against the planktonic and biofilm growth of each pathogen were determined, together with the minimum biofilm eradication concentrations (MBECs). Results revealed that *T. capitatus* EO was rich in thymol, *p*-cymene, and carvacrol, presenting high AA and TPC (144.66 μmol Trolox^TM^ equivalents and 231.32 mg gallic acid equivalents per g of EO, respectively), while its MICs and MBICs ranged from 0.03% to 0.06% *v*/*v* and 0.03% to 0.13% *v*/*v*, respectively, depending on the target pathogen. The EO was able to fully destroy preformed (mature) biofilms of all three pathogenic species upon application for 15 min, with MBECs ranging from 2.00 to 6.25% *v*/*v*. Overall, the results demonstrate that the EO of organically cultured *T. capitatus* presents strong antioxidant, antibacterial, and antibiofilm properties and could, therefore, be further exploited as a functional and antimicrobial natural formulation for food and health applications.

## 1. Introduction

Today, the resistance of microorganisms, mainly clinically important bacteria, to antimicrobials, including antibiotics that are important for human medicine, is a global challenge for humanity [1]. Indeed, this huge ecological problem has in recent years been further emphasized in the framework of the One Health concept aiming to sustainably balance and optimize the health of people, animals, and ecosystems [2]. It is now widely accepted that antimicrobial resistance not only threatens our ability to successfully treat human infections but is also strongly associated with activities and significant problems in many interrelated areas, such as animal husbandry, aquaculture, crops, food, and waste processing [3,4]. In the food industry, for instance, resistant bacteria (either to biocides used to disinfect processing equipment and/or preservatives added to foods to delay microbial growth) may cause important economic losses (by spoiling foods and shortening their shelf life), while those that are pathogenic may be implicated in foodborne disease outbreaks, thus endangering consumers’ well-being and public health in general [5,6].

Alarmingly, each year worldwide, unsafe food causes 600 million cases of foodborne diseases and 420,000 deaths, according to estimates from the World Health Organization [7]. In the United States alone, data from active and passive surveillance and other sources roughly calculate that each year 31 major pathogens cause 9.4 million foodborne illnesses, more than 55,000 hospitalizations, and more than 1300 deaths [8]. According to these data, nontyphoidal *Salmonella* spp. and *Listeria monocytogenes*, with some other microorganisms, are among the leading causes of hospitalization and death. However, salmonellosis and listeriosis are usually self-limiting gastrointestinal infections which do not require antimicrobial therapy [9]. Nevertheless, in susceptible individuals, such as infants, older adults, pregnant women, and those with a weakened immune system, these infections generally lead to hospitalization, while in the case of invasive listeriosis, the fatality rate may even exceed 25%. *Yersinia enterocolitica* is another emerging foodborne pathogenic bacterium that, together with *Salmonella* spp., belongs to the Enterobacteriaceae family [10]. Yersiniosis is a gastrointestinal infection that affects humans mainly through the ingestion of food, especially raw or undercooked pork [9]. Based on the latest available data for Europe, salmonellosis and yersiniosis were the second and third most-reported human zoonoses in 2020, exceeded only by campylobacteriosis [11]. It is worth noting that *Y. enterocolitica* and *L. monocytogenes* can both grow at refrigeration temperatures (i.e., at or below 4 °C) making their control more challenging [9].

Once found outside their hosts, *Salmonella* spp., *L. monocytogenes*, and *Y. enterocolitica*, like several other bacterial pathogens and microorganisms, can attach strongly to abiotic surfaces, such as those coming into contact with food, and form multicellular sessile structures called biofilms [12,13,14,15]. These surface-associated microbial communities are embedded in self-produced (or even acquired) hydrated extracellular polymeric matrixes and offer many advantages to their enclosed cells. The increased tolerance and resistance of these cells to biocides, as well as to various adverse environmental conditions, such as desiccation, pH extremes, and nutrient deprivation, are among the most important of these benefits [16,17]. On the other hand, biofilms formed by pathogens inside the human body are recalcitrant to both antibiotics and the immune system mechanisms and in this way are responsible for persistent (chronic) infections that represent a significant clinical challenge [18,19]. Alarmingly, pathogenic biofilm cells may not only be harder to eradicate, but those that survive may also present an increased virulence [20].

In recent years, issues such as these have prompted an increasing interest in the discovery, development, and application of novel, cost-efficient, and preferably eco-friendly (green) antimicrobial compounds and strategies to control unwanted biofilms in both industrial and clinical settings [21,22]. To this end, the use of plant extracts, such as essential oils (EOs) or other individual phytochemicals, has been explored either alone (e.g., thymol, carvacrol) or in combination with other compounds (e.g., antibiotics) or different antimicrobial approaches (e.g., bacteriophages, nanocoatings) [23,24,25,26,27]. EOs are particularly complex mixtures of volatile plant secondary metabolites, consisting mainly of terpenes, terpenoids, and phenylpropanoids [28]. These have been exploited by humans for thousands of years for various purposes (e.g., to add flavor to food or as ingredients in perfumes and cosmetics). They have also been used widely in folk medicine because of their many different bioactivities, such as antioxidant, antimicrobial, anti-inflammatory, antiparasitic, hepatoprotective, analgesic, and antitumor properties [29]. In recent years, the number of studies on the antibiofilm and/or antivirulence properties of various EOs has been increasing. It is interesting to note that such actions are often exhibited even upon their applications at very low concentrations (without any apparent effect on the growth of planktonic cells), something that together with their multitarget (non-specific) action is believed to limit the possibility for microbial cells to develop resistance [30,31].

Thyme is an aromatic and medicinal perennial wild shrub that belongs to the Lamiaceae family and is native to the Mediterranean region [32]. Interestingly, more than 900 species of the genus *Thymus* have been identified throughout the world, with *Thymus capitatus* (L.) being one of them. This latter is a synonym of *Coridothymus capitatus*, *Satureja capitata*, *Thymbra capitata*, and *Thymus creticus* and is more commonly known as conehead thyme, Persian hyssop, or Spanish oregano [33]. It is frequently used in traditional treatments of numerous diseases [34]. The present study examines the antibacterial and antibiofilm action of the EO of *T. capitatus*, extracted from plants organically cultured on the Greek Island of Lemnos (northeastern Greece), against *S. enterica* ser. Typhimurium, *L. monocytogenes*, and *Y. enterocolitica*. It is generally accepted that organic agricultural systems are more profitable and more environmentally friendly than conventional farming and deliver equivalent or higher-quality products that contain less (or no) pesticide residues [35]. While there are some previous antimicrobial and antibiofilm studies employing the EO from this aromatic and medicinal species [36,37,38,39,40,41,42,43,44], wild plants have most of the times been used to extract the oil. In addition, to the best of our knowledge, no other study has ever tested the action of *T. capitatus* EO against the biofilms of the aforementioned three important pathogens. In parallel, the antioxidant activity (AA) of the EO together with its total phenolic content (TPC) were also evaluated using some classical assays, while its chemical composition was determined through gas chromatography (GC) analyses (GC-FID and GC-MS).

## 2. Results and Discussion

The antimicrobial action of the EO was initially evaluated by determining its minimum inhibitory concentrations (MICs) and minimum bactericidal concentrations (MBCs) against the planktonic cells of each one of the three foodborne bacterial pathogens. Thus, for both *L. monocytogenes* and *Y. enterocolitica,* the MIC of the EO was equal to 0.03 *v*/*v* (0.3 mg/mL), while *S*. Typhimurium was slightly more resistant, with the MIC equal to 0.06% *v*/*v* (0.6 mg/mL) (Table 1). The absorbance curves (A_600 nm_) of each bacterial strain during its 24-h growth in Trypticase Soya Broth (TSB) at 37 °C in the presence of ten different *T. capitatus* EO concentrations (two-fold dilutions ranging from 0.50 to 0.001 % *v*/*v*) are shown in Figure S1. The MBC values were the same as the MIC values for *S*. Typhimurium and *Y. enterocolitica*, so the application of the EO at its MIC not only inhibited the growth of these strains but, at the same time, was sufficient to kill (by more than 99.9%) their planktonic cells. In the case of *L. monocytogenes*, the EO needed to be applied twice its MIC to kill (by more than 99.9%) pathogen’s planktonic cells (MBC = 0.06% *v*/*v* = 0.6 mg/mL) (Table 1).

The antimicrobial results (and especially the ratio MBC/MIC ≤ 2) demonstrate a strong bactericidal action of the EO against the planktonic cells of the tested pathogens and agree with the literature. A previous assessment of the in vitro antimicrobial activity of the EO extracted from *C. capitatus* growing wild in Lebanon against the yeast *Candida albicans* and six pathogenic bacteria (including *Escherichia coli*, *Pseudomonas aeruginosa*, *Enterococcus faecalis*, *S. enterica* ser. Enteritidis, *Staphylococcus aureus* and *Bacillus subtilis*) using the broth dilution method resulted in MIC values ranging from 0.2 to 0.8 mg/mL, depending on the microbial species [45]. Faleiro et al. tested the antibacterial action of *T. capitata* EO from Portugal against 12 *L. monocytogenes* strains and found that MIC values ranged from 0.05 to 0.20 μL/mL (equal to 0.005 to 0.02% *v*/*v*), while the MBC was equal to 0.30 μL/mL (0.03% *v*/*v*) for all tested strains [46]. In another study that evaluated the antibacterial action of Libyan wild-growing *T. capitatus* EO against eight bacterial species (four Gram-negative including *S*. Typhimurium and four Gram-positive including *L. monocytogenes*), the results indicated an extremely high antibacterial effect of the EO with the MIC varying between 1–2 μg/mL and MBC between 1–40 μg/mL [47]. Peñalver et al. determined the MIC values of *C. capitatus* EO from Spain against four *S. enterica* strains (of different serotypes, including Enteritidis and Typhimurium) and found that these ranged between 0.5 to 4.0% *v*/*v* [48]. Tagnaout et al. tested the antibacterial action of *T. capitata* EO from Morocco against eight bacterial species (*E. faecalis*, *Serratia fonticola*, *Acinetobacter baumannii*, *Klebsiella oxytoca*, *Klebsiella pneumoniae*, *E. coli*, *S. aureus*, and *Enterobacter aerogenes*) and found that MIC values ranged from 2 to 16 μL/mL (equal to 0.2 to 1.6% *v*/*v*). In that older study, the MBC values were in general twice the MIC values and ranged from 4 to 32 μL/mL (equal to 0.4 to 3.2% *v*/*v*) [49]. Moumni et al. determined the MIC values of Tunisian *T. capitatus* EO against four bacterial species (including *P. aeruginosa*, *E. coli*, *S. enterica*, *B. subtilis*, and *S. aureus*) and found that they ranged between 0.73 and 2.94 mg/mL [50]. Considering previous results, it is concluded that the *T. capitatus* EO studied here presents an antibacterial efficiency that is equivalent to or several times higher than that of EOs extracted from wild plants of the same species growing in some other countries and can kill the planktonic cells of the three tested pathogens in concentrations even less than 1 mg/mL (0.1% *v*/*v*).

**Figure 1:**
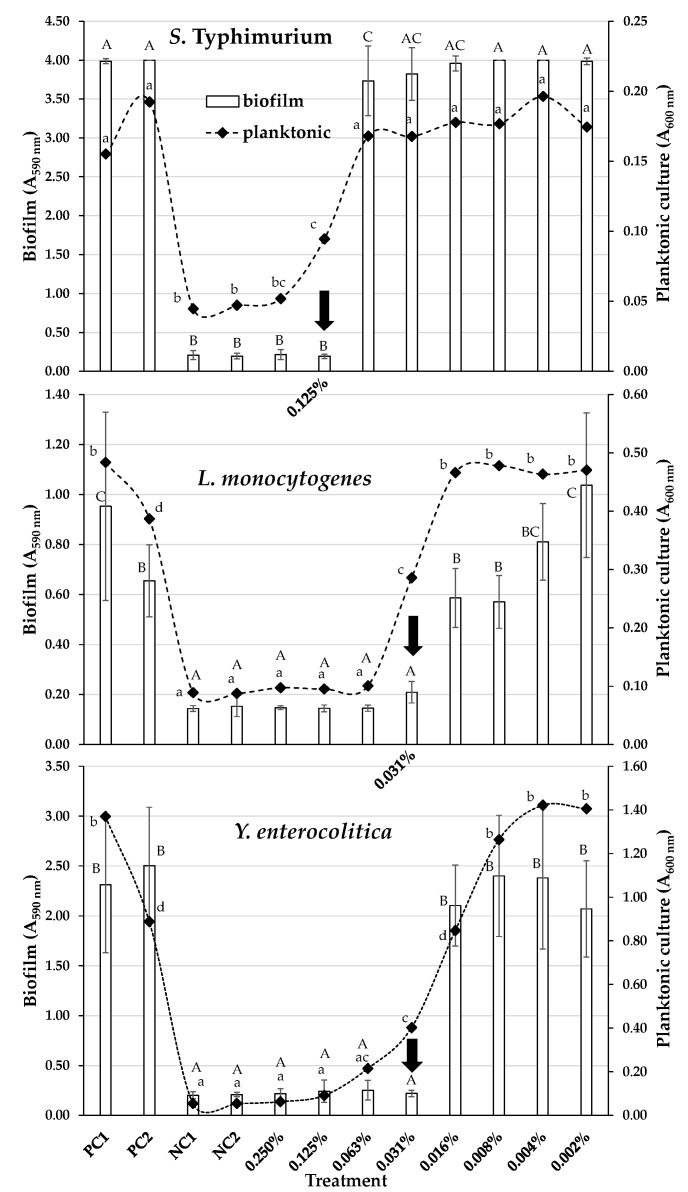
Biofilm formation (A_590 nm_) by *S*. Typhimurium, *L. monocytogenes*, and *Y. enterocolitica* strains on the PS surface of the 96-well microtiter plates, in the presence of eight different *T. capitatus* EO concentrations (two-fold dilutions ranging from 0.25 to 0.002 % *v*/*v*). The bars represent the mean values ± standard deviations. The accumulated biofilm biomasses for two positive (PC1, PC2) and two negative controls (NC1, NC2) are also shown. For each bacterial strain, the MBIC of the EO (resulting in the total inhibition of biofilm formation) is indicated by the vertical arrow. The absorbances of planktonic suspensions (A_600 nm_) found in the same wells at the time of sampling (96 h) are also shown for each treatment (as rhombuses joined by dotted curved lines). The bars of standard deviations of the planktonic means were omitted for clarity. In each graph (bacterial strain), mean biofilm values followed by different superscript uppercase letters (ABC) differ significantly (*p* < 0.05). In each graph, mean planktonic values followed by different superscript lowercase letters (abcd) differ significantly (*P* < 0.05).

After testing the antibacterial action of the EO against the planktonic pathogenic bacteria, we evaluated its action against biofilm formation of the same pathogens by calculating the minimum biofilm inhibitory concentrations (MBICs), which are defined as the lowest EO concentrations that prevented biofilm formation of each tested strain. For both *L. monocytogenes* and *Y. enterocolitica,* the MBIC of the EO was equal to 0.03 *v*/*v* (0.3 mg/mL), while for *S*. Typhimurium, an almost triple concentration was required to suspend biofilm formation with the MBIC equal to 0.13% *v*/*v* (1.1 mg/mL) (Table 1). Figure 1 shows the biofilm quantities (given as total sessile biomasses; A_590 nm_) for each one of the three tested pathogens on the wells of the polystyrene (PS) microtiter plates and in the presence of the eight different *T. capitatus* EO concentrations that were tested (two-fold dilutions ranging from 0.25 to 0.002 % *v*/*v*). The absorbances of planktonic suspensions (A_600 nm_) found in the same wells at the time of sampling (96 h) are also shown for each treatment (as an indirect indication of the concentrations of planktonic populations). It is observed that the incubation of all three bacterial strains together with the respective MBIC of the EO resulted in the total inhibition of biofilm formation (biomass accumulated was not significantly different from the negative controls). However, a significant population of planktonic cells was found in the same wells (which differed from that of the negative controls), indicating that bacteria were still able to multiply (although to a lesser extent than the positive controls that did not contain any EO) without being capable of creating a biofilm. This is interesting because it denotes that the *T. capitatus* EO, besides its antibacterial action, should present some antibiofilm-specific mechanism(s) against the target cells (e.g., inhibition of cell-to-cell aggregation, interference with intercellular communication, inhibition of bacterial attachment to the substratum, modification of the extracellular polymeric matrix, and loss of its coherence) at sub-MIC values. Indeed, this has also been observed in some previous antibiofilm studies with other plant extracts and phytochemicals [27,51,52,53].

It should be noted that given the different experimental conditions of the two assays (with respect to the growth medium, incubation time, and temperature), it is not possible to directly compare the MIC results retrieved from the broth microdilution assays for the planktonic cells (Table 1) and the minimum EO concentrations that inhibited the proliferation of planktonic cells in the wells of the MBIC assays (Figure 1). Thus, for the determination of MICs, all bacteria were grown for 24 h at 37 °C in TSB under optimum planktonic growth conditions, while for the determination of MBICs, bacteria were left to develop biofilms on the PS microtiter plates for 96 h under strain-specific biofilm-maximizing conditions (see Section 3.5).

**Figure 2:**
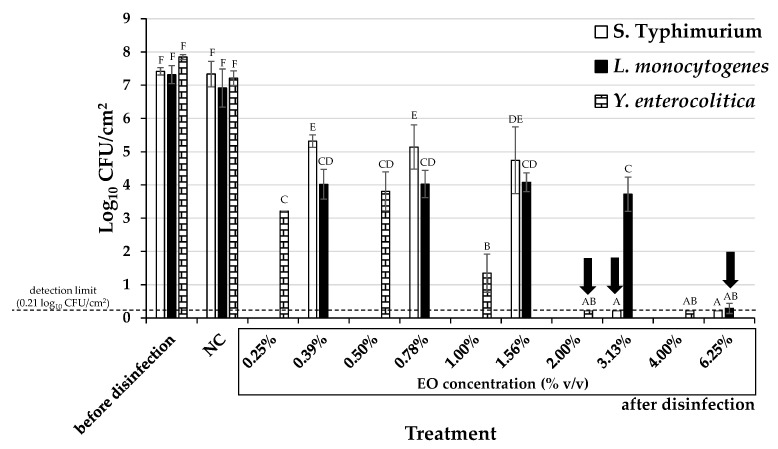
Biofilm populations (log_10_ CFU/cm^2^) found in the wells of the 96-well microtiter plates for the three bacterial strains (*S*. Typhimurium, *L. monocytogenes*, and *Y. enterocolitica*) before and after disinfection with the *T. capitatus* EO. For each bacterial strain, the EO was tested in five different concentrations, two-fold dilutions ranging from 0.39 to 6.25% *v*/*v* for *S*. Typhimurium and *L. monocytogenes* strains, and from 0.25 to 4.00% *v*/*v* for *Y. enterocolitica* strain. The biofilm population of the negative disinfection control (NC; sterile distilled water also containing 10% *v*/*v* ethanol) is also shown. The bars represent the mean values ± standard deviations. Mean values followed by different superscript uppercase letters (A–F) differ significantly (*p* < 0.05). For each bacterial strain, the MBEC of the EO (resulting in more than six log reductions of its biofilm population with respect to that of the NC) is indicated with the vertical arrow.

Following the calculation of the MBICs of the EO against biofilm development, the EO was also tested against preformed (mature) biofilms of the selected strains. For this, biofilms were allowed to form under the optimum conditions for each strain and were then disinfected (for 15 min) by applying a range of different EO concentrations (from 0.25 to 6.25% *v*/*v*). For each treatment, the biofilm cells that survived disinfection were detached from surfaces (through scratching) and enumerated by agar plate counting. The derived biofilm eradication results are presented in Figure 2, while the minimum biofilm eradication concentrations (MBECs) of the EO against each bacterial strain are shown in Table 1. Thus, in order to kill biofilm cells (to cause about six log reductions considering the initial sessile concentrations), the EO needed to be applied from 52.2 times more than its MBC against *S*. Typhimurium to 104.2 times more than its MBC against *L. monocytogenes*. In the case of *Y. enterocolitica*, the determined MBEC (2% *v*/*v*) was 66.7 times more than its MBC against the planktonic cells of the same strain. Such huge (or even higher) increases in effective biocide concentrations against biofilm cells in relation to their effective concentrations against planktonic cells have been observed in numerous other studies [53,54,55] and accord with the great resistance that mature biofilms are known to present [16,17]. Nevertheless, the fact that the *T. capitatus* EO could eradicate the formed biofilms of all three strains (detection limit of the plate counting method: 0.21 log_10_ CFU/cm^2^) after a short exposure (15 min) at a concentration of less than 7% *v*/*v* still demonstrates its strong antibiofilm action. It is worth noting that all three strains reached (on the fourth day of incubation and before disinfection) sessile concentrations (on the PS surface) that exceeded 7 log_10_ CFU/cm^2^ (Figure 2). For *S*. Typhimurium, the quantification of its total sessile biomass coincided in parallel with the detection plateau of the microplate absorbance reader (A_590 nm_ = 4) (Figure 1), indicating a very rich amount of negatively charged extracellular substances (e.g., polysaccharides) able to bind the added cationic dye (crystal violet; CV).

To the best of our knowledge, no other studies testing the antibiofilm efficiency of *T. capitatus* EO against these three bacterial pathogens have ever been undertaken. In a similar study, Alabdullatif et al. tested the biofilm-killing efficiency of three concentrations of Tunisian *T. capitatus* EO (10, 20, and 30% *v*/*v*) against *Staphylococcus epidermidis* biofilms (preformed on 96-well PS microtiter plates at 37 °C for 24 h) following 30 s of application (to mimic skin-disinfection practices), and they observed log reductions that ranged from 1.17 ± 0.12 to 1.42 ± 0.10 log_10_ CFU/mL [36]. Almeida et al. tested the biofilm-disrupting efficiency of *T. capitata* EO collected from plants grown in the south of Portugal against preformed biofilms (on 96-well PS microtiter plates at 37 °C for 24 h) of six clinical methicillin-resistant *S. aureus* (MRSA) isolates by exposing them for 24 h (at 37 °C) to 0.64 μg/mL of the EO (a concentration that was equal to its MBC) [37]. The action of the EO in that study was also compared with the action of two antibiotics with distinct mechanisms of action and commonly used to treat MRSA infections (ciprofloxacin and tetracycline) and tested at their respective peak serum concentrations [37]. The biofilm cells of all tested isolates were significantly affected in their culturability and metabolic activity after incubation with the EO, with an observed average reduction of 2.6 log_10_ CFU/mL, significantly higher than that observed with the tested antibiotics (for both, an average reduction of less than of 0.5 log_10_ CFU/mL was observed). However, the EO was still insufficient to significantly reduce the total biofilm biomass of four of the six isolates (as this was quantified through CV staining). 

Within the realm of research that has also examined the biofilm-disrupting capacity of *T. capitatus* EO, Machado et al. evaluated the activity of Portuguese *T. capitata* EO against *Gardnerella vaginalis* biofilm through quantification of biomass removal efficiency (by CV staining) [39]. For this, seven clinical strains were individually left to form biofilms on 96-well PS microtiter plates (at 37 °C for 48 h under 10% CO_2_) and then exposed for 24 h to three different EO concentrations (0.16, 0.32, and 0.64 μL/mL; equal to 0.016, 0.032, and 0.064% *v*/*v*). *Thymus capitata* EO at 0.64 μL/mL (equal to 3 x MBC) was effective against all biofilms tested, provoking percentages of biofilm biomass reduction that ranged between 49 and 91%, also visualized and confirmed by confocal laser scanning microscopy (CLSM). However, survival rates of the remaining enclosed biofilm cells were not determined in that older study. In two other similar studies by the same group, this EO was also found to present an important biomass removal effect on preformed *Candida* spp. biofilms (of 13 clinical isolates that belonged to four different species) [40], as well as a preformed polymicrobial biofilm that consisted of six cultivable bacterial vaginosis-associated bacterial species [41]. In another study, the EO from Italian *C. capitatus* at 1% *v*/*v* was found to inhibit biofilm formation and reduce preformed biofilms in about half of eleven clinical *P. aeruginosa* isolates from cystic fibrosis patients [42]. Scanning electron microscopy (SEM) analysis also revealed that this EO treatment resulted in a dramatic modification of the extracellular matrix biofilm structure. At the same time, this drastically attenuated some other important virulence properties of almost all tested isolates (pyocyanin production, swimming, and swarming motilities).

For the determination of the antioxidant activity (AA) of *T. capitatus* EO, two methods were here performed (DPPH^•^ and CUPRAC assays), while for the evaluation of its total phenolic content (TPC), the Folin–Ciocalteu method was conducted. The results of these three methods are presented in Table 2.

The EO of *T. capitatus* presented high AA in both DPPH^•^ and CUPRAC assays. High AA of *T. capitatus* EO as well as some other extracts of this species has been confirmed by numerous research results [34]. For instance, in some other comparative analyses, *T. capitatus* EO has been found to induce the highest AA when compared with EOs from other thyme species [56] and those from some other species of the same family (Lamiaceae), such as *Clinopodium suaveolens*, *Satureja montana*, and *Salvia fruticosa* [57]. However, based on the DPPH^•^ assay, our results (144.66 ± 1.71 μmol TRE/g EO; equal to 32.9 mg TRE/mL EO) demonstrate an AA that is similar to that previously observed with *Thymus vulgaris* EO (149.8 ± 6.7 μmol TRE/g EO) though the TPC of *T. capitatus* EO from Lemnos (231.32 ± 16.71 mg GAE/g EO) was higher than that of the *T. vulgaris* EO of that older study (177.3 ± 1.9 mg GAE/g EO) [58]. In another study, the EO derived from *T. capitatus* plants of two sampling areas in Apulia, Italy, was found to present an AA equal to 9.61 ± 0.19 and 8.39 ± 0.11 mg TRE/mL EO when tested by the DPPH^•^ assay [57], with both those values being lower than that recorded here by the same assay (32.9 mg TRE/mL EO). The high AA of *T. capitatus* EO has been attributed to the presence of several classes of bioactive compounds, including flavonoids, terpenoids, and phenolic acids, with its TPC being extensively linked to that property [34]. The results of the present study show that the TPC of *T. capitatus* EO is very high (231.32 mg GAE/g EO; equal to 210.48 mg GAE/mL EO), approaching the highest values of TPC that have been reported for EOs derived from Italian *T. capitatus* plants (279–286 mg GAE/mL EO) [57]. This high TPC should also be connected to the high antimicrobial activity of the EO we observed given that several phenolic compounds are well-known to exert antimicrobial action [59]. In another study, the EO from *T. capitatus* plants collected from northern Morocco presented a TPC equal to 5.18 ± 0.68 mg GAE/g EO [60]. 

Such discrepancies in the AA and TPC results between different studies are probably explained by the diversity in the chemical composition of the EOs extracted from even the same species. The chemical composition is indeed known to be affected by the particular soil and climate conditions of each region, as well as the plant organ, age, vegetative cycle stage, and method used to extract the oil [32,61,62]. The chemical composition of the studied here *T. capitatus* EO, as revealed through its GC-FID and GC-MS analyses, is presented in Table 3. It was mainly composed of thymol, *p*-cymene, carvacrol, *γ*-terpinene, limonene, linalool, and *α*-pinene; twenty-two other compounds were also identified (GC-MS), representing 99.2% of the total oil content. It should be noted that all twelve compounds that were identified through the GC-FID analysis were also detected by the GC-MS analysis in similar peak area percentages (%). Most of these components are usually detected in EOs extracted from this species, which is usually primarily composed of carvacrol [34]. However, thymol has also been detected as the main component of *T. capitatus* EO in some other studies [63,64,65]. Both thymol and carvacrol are terpenoids included in the most bioactive phytochemicals isolated from EOs, frequently being the main components occurring in the EOs isolated from plants of the Lamiaceae family [66,67].

## 3. Materials and Methods

### 3.1. Chemicals

The crystal violet (CV) solution (1% w/v, aqueous solution), 2,2-diphenyl-1-picrylhydrazyl (DPPH), 6-hydroxy-2,5,7,8-tetramethylchromane-2-carboxylic acid (Trolox^TM^), neocuproine, ammonium acetate, gallic acid, and hexane were all purchased from Sigma-Aldrich Chemie GmbH (Taufkirchen, Germany). Ethanol (EtOH), methanol (MeOH), Folin–Ciocalteu reagent, and sodium carbonate monohydrate (Na_2_CO_3_·H_2_O) were obtained from Merck (Darmstadt, Germany). Copper (II) chloride hydrate (Puratronic^®^, 99.999%) was provided by Thermo Fisher GmbH (Kandel, Germany).

### 3.2. Hydrodistillation and Receipt of EO

The EO of organically cultivated *T. capitatus* plants (Aegean Organics, Agios Dimitrios, Lemnos, Greece) was distilled in a 110 L stainless steel (18/10) distiller. A perforated basket containing 5 kg (dry weight) of the top cuttings of thyme (stems, leaves, and flowers) was loaded in the distillation rig followed by the addition of 10 L of water. The distillation time was 4 h and yielded 1.1% *v*/*w* EO. The oily fraction of the resultant product was collected in a 500 mL conical, graduated separating funnel equipped with a polytetrafluoroethylene (PTFE) stopcock. Immediately after the distillation, the conical funnel and its contents were put in the freezer (−20 °C) for 24 h to achieve complete separation from the water. The resultant EO was kept sealed in a dark amber glass in the fridge (4–6 °C) until further analysis.

### 3.3. Bacterial Strains and Preparation of Their Working Cultures

The bacterial strains used in this work belonged to the species *S. enterica*, *L. monocytogenes*, and *Y. enterocolitica* (one strain/species). Some critical information on these strains is provided in Table 4. Before their experimental use, these were stored long-term at −80 °C in BHI broth (Lab M, Heywood, Lancashire, UK) containing 15% *v*/*v* glycerol. When needed for the assays, each strain was streaked on the surface of Tryptone Soya Agar (TSA; Oxoid, Thermo Fisher Specialty Diagnostics Ltd., Hampshire, UK) and incubated at 37 °C for 24 h (preculture). Working cultures were prepared by inoculating an individual colony from each preculture into 10 mL of fresh TSB (Condalab, Torrejón de Ardoz, Madrid, Spain) and then incubating at 37 °C for 24 h (thereby achieving a final concentration of ca. 10^9^ CFU/mL). The purity of each working culture was always confirmed by streaking a small volume of it (10–20 μL) on TSA and incubating it at 37 °C for 24–48 h.

### 3.4. Determination of Minimum Inhibitory and Bactericidal Concentrations of EO against Planktonic Bacteria (MICs, MBCs)

The MIC of the EO against the planktonic growth of each of the three bacterial strains was determined using the broth microdilution method in a Bioscreen C° Pro instrument (Oy Growth Curves Ab Ltd., Turku, Finland). For this, a stock solution of the EO at 1% *v*/*v* was initially prepared on the day of the experiment in TSB also containing 9% *v*/*v* EtOH to help in the solubilization of the EO. Starting with this stock solution, two-fold dilutions were executed in TSB to prepare ten different EO concentrations ranging from 0.5 to 0.001% *v*/*v*. Samples of 360 μL of each of these dilutions were then placed in duplicate in each well of a special 100-well Bioscreen honeycomb plate (Product code # 95025BIO) and inoculated with 40 μL of a 1:1000 dilution (in TSB) of the working culture of each strain (to start with an initial concentration of ca. 10^5^ CFU/mL). The plate was subsequently incubated at 37 °C for 24 h in the heated chamber of the Bioscreen C° Pro instrument, which was set to record the absorbance of the contents of each well at 600 nm (A_600 nm_) every 30 min. Prior to each measurement, the plate was automatically shaken for 5 s. For each bacterial strain (*n* = 3) and EO concentration (*n* = 10), a growth curve (A_600 nm_ *vs* time) was automatically generated by the BioScreener PRO Software, while the data of all the growth curves were finally exported to the Excel^®^ module of the Microsoft^®^ Office 365 suite (Redmond, WA, USA). As a positive control for bacterial growth, inoculated growth medium was used that also contained 0.6% *v*/*v* EtOH. This latter concentration was the one existing in the higher MIC value recorded, i.e., 0.06% *v*/*v* EO, as this was revealed against *S*. Typhimurium in some preliminary experiments. Pure sterile growth medium (TSB) was used as a negative control. For each bacterial strain, the MIC of the EO was determined as its lowest concentration resulting in no visible growth (no increase in broth’s absorbance with respect to the negative control during whole incubation). To calculate the MBC, 10 μL of broth cultures were aspirated from all the non-growth wells of the MIC assay and spotted (in duplicate) on TSA plates, which were then incubated at 37 °C for 24–48 h. For each bacterial strain, the MBC of the EO was determined as its lowest concentration that reduced the initial inoculum (*ca*. 10^5^ CFU/mL) by more than 99.9% (no appearance of colonies). Each of these experiments was repeated three times starting with independent bacterial cultures.

### 3.5. Determination of Minimum Biofilm Inhibitory Concentrations (MBICs)

The MBIC of the EO against the biofilm growth of each strain was determined by the CV staining assay as previously described [85]. For this, bacteria were initially left to form biofilms on 96-well PS microtiter plates (transparent, flat, Cat. No. 30096, SPL Life Sciences, Gyeonggi-do, Korea) for 96 h in the appropriate growth medium and temperature depending on the strain (Table 5) and in the presence of eight different concentrations of the EO (two-fold dilutions ranging from 0.25 to 0.002% *v*/*v*). In all cases, a renewal of growth media took place at 48 h. At the end of incubation, for each bacterial strain and EO concentration, accumulated biomasses in each well were quantified following their staining with CV (0.1% (*w*/*v*)), solubilization of the bound dye with an ethanol-acetone mixture (80:20, *v*/*v*), and absorbance measurements of the resulting solutions at 590 nm (A_590 nm_) using a multimode microplate reader (Tecan Spark^®^, Tecan Group Ltd., Männedorf, Switzerland). The absorbances of the planktonic suspensions (A_600 nm_) found in the wells at the time of sampling (96 h) were also recorded just before their removal and the subsequent double-washing of the wells with quarter-strength Ringer’s solution (Lab M) to remove the loosely attached cells. Two positive controls (PC) were used in parallel for biofilm growth. In one of them, the inoculated with bacteria medium (*ca*. 10^5^ CFU/mL) did not contain any EO (PC1), while in the other (PC2) the inoculated growth medium was supplemented with 2.25% *v*/*v* EtOH. This concentration existed in the broths containing the maximum EO concentration that was tested (0.25% *v*/*v*). In parallel with the two positive controls, two negative controls were also employed (NC1 and NC2), in both of which no bacteria were added. For each bacterial strain, the MBIC of the EO was determined as its lowest concentration that completely inhibited biofilm formation (biomass accumulated was not significantly different from the negative controls). Each experiment was repeated at least three times starting with independent bacterial cultures.

### 3.6. Determination of Minimum Biofilm Eradication Concentrations (MBECs)

The MBEC of the EO against the already 96 h preformed biofilms of each bacterial strain was determined by following a previously described protocol [52] with some adaptations which are reported below. In brief, following biofilm formation (again under the optimum biofilm-forming conditions for each strain, see Table 5) and the removal of the surrounding planktonic suspensions and loosely attached cells, 200 μL of a range of different EO solutions were added in quadruplicate to each well and left in contact for 15 min at room temperature (disinfection/eradication step). The EO was tested in five different concentrations, two-fold dilutions ranging from 6.25 to 0.39% *v*/*v* for *S*. Typhimurium and *L. monocytogenes* strains, and from 4.00 to 0.25% *v*/*v* for *Y. enterocolitica* strain, which were all prepared in sterile distilled water (dH_2_O), always starting from the same stock solution (10% *v*/*v* EO, 16% *v*/*v* EtOH, 74% *v*/*v* sterile dH_2_O). Sterile dH_2_O also containing 10% *v*/*v* EtOH was used as the negative disinfection control (NC). This EtOH concentration was the maximum one existing in the 6.25% *v*/*v* EO working disinfection solution. Following disinfection, the antimicrobial solutions were carefully removed from each well, and 250 μL of Dey-Engley (D-E) Neutralizing broth (Lab M) was then added and left in contact for 15 min. After the neutralization step, the submerged surface of each well was thoroughly scratched with a plastic pipette tip to remove the biofilm bacteria. Following the scratching, the (identical) suspensions of the four replicate wells were combined in a single Eppendorf^®^ tube (1 mL) and swirled for 1 min at maximum speed (3200 rpm) using a mini vortex mixer (VXMNAL, Ohaus Europe GmbH, Nänikon, Switzerland) to disrupt any cellular aggregate. After that, the mixed bacterial suspension was serially diluted (1:10 till 10^−6^) in quarter-strength Ringer’s solution and spread-plated (100 μL) on duplicate TSA plates. Bacterial colonies were counted following incubation at 37 °C for 48 h, and their numbers were converted to CFU/mL and subsequently to log_10_ CFU/cm^2^ (considering that the total surface area of each well initially covered by the 200 μL of the inoculated growth medium was equal to 1.53 cm^2^). For each bacterial strain, the MBEC of the EO was determined as the lowest concentration at which it provoked at least six log reductions of the biofilm population compared to that of the NC. For each bacterial strain, the population of the biofilm bacteria (log_10_ CFU/cm^2^) found in the wells at the end of the 96 h incubation, after the removal of the loosely attached cells, and before the disinfection was also quantified through the same scratching and agar plating procedures. Each experiment was repeated at least three times starting with independent bacterial cultures.

### 3.7. Determination of the Antioxidant Activity (AA) of the EO

#### 3.7.1. DPPH^•^ Assay

For the determination of the DPPH^•^ scavenging activity, a previously described protocol was followed [86]. For this, the EO was initially diluted 1:100 *v*/*v* in MeOH. Results were finally expressed as μmol TRE/g EO (considering the density of the EO was equal to 0.91 g/mL). All the measurements were repeated five times.

#### 3.7.2. Cupric Ion Reducing Antioxidant Capacity (CUPRAC) Assay

The Cu (II) reducing capacity of the EO was measured according to a previously described protocol [87]. In brief, 1 mL of a 0.02 mol/L solution of copper (II) chloride, 1 mL of a 7.5 mmol/L neocuproine solution, and 1 mL of a 1 mol/L ammonium acetate buffer (pH = 7.0) were mixed with 25 μL of a diluted methanolic solution (1:100 *v*/*v*) of the EO. After adding dH_2_O (to a final volume of 4.1 mL), the mixture was shaken for 15 s. The absorbance at 450 nm was measured using a UV-Vis spectrophotometer (UV-1800, Shimadzu Co., Kyoto, Japan) after the solution had been allowed to stand in the dark for 30 min. The results were finally expressed as μmol TRE/g EO after correction with an appropriate blank. The repeatability of measurements was calculated for a standard solution of Trolox^TM^ and was found to be satisfactory (coefficient of variance = 4.3%). All the measurements were repeated five times.

### 3.8. Determination of the Total Phenolic Content (TPC) of the EO

The TPC of the EO was evaluated using the Folin–Ciocalteu method, as previously described [86]. For this, the EO was initially diluted 1:100 *v*/*v* in MeOH. Results were finally expressed as mg GAE/g EO. All the measurements were repeated five times.

### 3.9. Chemical Analysis of EO (GC)

#### 3.9.1. GC-FID

This analysis was performed for a fee in an external (private) analysis laboratory (Food Allergens Laboratory, Nea Ionia, Greece). This was done on an Agilent 6890N (G1540N; Agilent Technologies, Santa Clara, CA, USA) GC instrument equipped with a flame ionization detector (FID) and an Rtx-mineral oil column (length 15 m × 0.32 mm ID, 0.10 µm df; Restek, United States). Helium (3.0 mL/min, avg: 89 cm/s) was used as carrier gas and the oven temperature program used a complex gradient of 50–110 °C (88.75 min) and 110–300 °C (5 min) with 10 min post run at 300 °C. Sample (1 µL) was injected at 150 °C front inlet temperature, splitless. Calculation of peak area percentage was performed based on the FID signal using the GC HP-Chemstation software (Agilent Technologies). The constituents were identified by comparison of their retention indices with those in the literature and the use of external standards of the identified compounds. Component relative percentages were calculated based on GC peak areas without using correction factors.

#### 3.9.2. GC-MS

The EO was diluted in pure hexane (1:9), and the mixture was then chemically characterized by GC-MS analysis on a 6890N, 5973NetworkedMS MSD system (Agilent Technologies) equipped with an HP-5MS column (30 m, 0.25 mm inner diameter, 0.25 μm film thickness) (Agilent Technologies), as previously described [88]. EO compounds were identified by comparing the retention times and mass spectra to NBS75K and Wiley275 reference libraries and in-house libraries, and by determining Linear retention indexes (LRIs) [89].

### 3.10. Statistics

The derived data on total biofilm biomasses (A_590 nm_), planktonic absorbances (A_600 nm_), and biofilm populations (log_10_ CFU/cm^2^) for each of the different treatments of the MBIC and MBEC assays were all submitted to analyses of variance (ANOVA), followed by Tukey’s multiple range post hoc honestly significant difference (HSD) tests for mean comparison, using the statistical software STATISTICA^®^ (StatSoft Inc., Tulsa, OK, USA). All differences are reported at a significance level of 0.05.

## 4. Conclusions

The EO of *T. capitatus*, a thyme species organically cultured on the Greek Island of Lemnos, was found to be rich in thymol, *p*-cymene, and carvacrol. It presented high AA and TPC values and the ability to prevent both planktonic and biofilm growth of three important foodborne pathogenic bacteria in very low concentrations (less than 0.1–0.2% *v*/*v*). In addition, the EO was able to fully destroy preformed (mature) biofilms of all three pathogenic species after a short exposure (15 min) at a concentration of less than 7% *v*/*v*. This could be further exploited as a functional and antimicrobial natural formulation for food and health applications.

## Figures and Tables

**Table 1:** Antibacterial (MIC and MBC values) and antibiofilm (MBIC and MBEC values) actions of *T. capitatus* EO against *S*. Typhimurium, *L. monocytogenes*, and *Y. enterocolitica* strains expressed as either EO percentages by volume (% *v*/*v*) or mg/mL.

Bacterial Species	Antibacterial Action				Antibiofilm Action			
	MIC ^1^		MBC ^2^		MBIC ^3^		MBEC ^4^	
	% (*v*/*v*)	mg/mL	% (*v*/*v*)	mg/mL	% (*v*/*v*)	mg/mL	% (*v*/*v*)	mg/mL
*S.* Typhimurium	0.06	0.6	0.06	0.6	0.13	1.1	3.13 (= 52.2 × MΒC)	28.4
*L. monocytogenes*	0.03	0.3	0.06	0.6	0.03	0.3	6.25 (= 104.2 × MΒC)	56.9
*Y. enterocolitica*	0.03	0.3	0.03	0.3	0.03	0.3	2.00 (= 66.7 × MΒC)	18.2

^1^ Minimum Inhibitory Concentration; ^2^ Minimum Bactericidal Concentration; ^3^ Minimum Biofilm Inhibitory Concentration; ^4^ Minimum Biofilm Eradication Concentration.

**Table 2:** Antioxidant activity (DPPH^•^ and CUPRAC assays) and total phenolic content (TPC) of *T. capitatus* EO (mean values ± standard deviations).

Method	DPPH^•^ (μmol TRE ^1^/g EO)	CUPRAC (μmol TRE ^1^/g EO)	TPC (mg GAE ^2^/g EO)
Results	144.66 ± 1.71	763.90 ± 13.93	231.32 ± 16.71

^1^ Trolox^TM^ equivalents; ^2^ Gallic acid equivalents.

**Table 3:** Chemical composition of *T. capitatus* EO as revealed by the GC-FID and GC-MS analyses.

Compounds Detected	GC-FID	GC-MS		
	% Area	LRΙ	LRIref	% Area
methyl-cyclopentane		<700	<700 [68]	0.8
Cyclohexane		<700	<700 [69]	0.1
α-Thujene		930	930 [70]	0.2
α-Pinene	2.9	936	937 [70]	2.8
Camphene		949	951 [70]	1.6
β-Pinene	1.7	977	978 [70]	0.3
β-Myrcene	1.5	999	992 [71]	1.0
α-Phellandrene		1008	1006 [72]	0.2
3-Carene		1012	1015 [73]	<0.1
α-Terpinene (1-methyl-4-(1-methylethyl)-1,3-cyclohexadiene)	0.2	1020	1016 [74]	0.4
Carvomenthene (1-methyl-4-(1-methylethyl)-cyclohexene)		1024	-	0.1
p-Cymene (1-methyl-4-(1-methylethyl)-benzene)	28.9	1034	1030 [75]	31.0
Limonene	4.2	1035	1033 [75]	3.9
p-Cymenene (1-methyl-4-(1-methylethenyl)-benzene)		1044	1078 [72]	0.1
γ-Terpinene (1-methyl-4-(1-methylethyl)-1,4-cyclohexadiene)	2.8	1070	1063 [73]	4.3
β-Thujone ([1S-(1α,4β,5α)]-4-methyl-1-(1-methylethyl)-bicyclo[3.1.0]hexan-3-one)		1114	1116 [74]	0.1
Linalool (3,7-dimethyl-1,6-octadien-3-ol)	5.1	1123	1121 [76]	3.8
Camphor		1154	1152 [77]	0.2
β-Terpineol (1-methyl-4-(1-methylethenyl)-cyclohexanol)		1169	1179 [76]	<0.1
Isoborneol		1173	1164 [78]	<0.1
Borneol		1183	1174 [73]	0.9
Terpinen-4-ol (4-methyl-1-(1-methylethyl)-3-cyclohexen-1-ol)		1191	1187 [71]	0.3
α-Terpineol (α,α-4-trimethyl-3-cyclohexene-1-methanol)	0.9	1209	1207 [79]	0.2
γ-Terpineol (1-methyl-4-(1-methylethylidene)-cyclohexanol)		1222	1218 [80]	<0.1
Thymol	44.5	1341	1302 [74]	39.8
Carvacrol (2-methyl-5-(1-methylethyl)-phenol)	4.2	1346	1311 [74]	5.7
Caryophyllene	1.0	1439	1437 [81]	1.1
α-Caryophyllene		1475	1465 [81]	0.1
Caryophyllene oxide		1610	1595 [71]	0.2
**Total**	97.9			99.2

LRI: Linear retention index.

**Table 4:** Bacterial strains used in this study and their relevant info.

Bacterial Species	Gram Reaction	Strain Code	Isolation Origin	Other Strain Information	Reference
*Salmonellla enterica*	*-*	FMCC ^1^_B137	human, salmonellosis outbreak	serovar Typhimurium, phage type DT193	[82]
*Listeria monocytogenes*	*+*	AAL ^2^ 20107	mixed green salad	serovar 1/2b	[83]
*Yersinia enterocolitica*	*-*	DSM ^3^ 4780	human, glanders-like infection of the face	subsp. *enterocolitica*, type strain, ATCC ^4^ 33114	[84]

^1^ Food Microbiology Culture Collection, Laboratory of Microbiology and Biotechnology of Foods, Department of Food Science and Human Nutrition, Agricultural University of Athens in Athens, Greece; ^2^ Athens Analysis Laboratories SA, Microbiology Laboratory in Metamorfosi, Greece; ^3^ DSMZ-German Collection of Microorganisms and Cell Cultures GmbH, Leibniz Institute in Braunschweig, Germany; ^4^ American Type Culture Collection, LGC Standards GmbH, Wesel, Germany.

**Table 5:** Biofilm-forming conditions that were applied for each bacterial strain. These were determined (in some preliminary experiments) as the ones maximizing biofilm growth by each strain following testing of all possible combinations (*n* = 12) of three different growth media (TSB, 1/10 TSB, and BHI broth), of two incubation temperatures (20 and 37 °C) and with the renewal or no renewal of growth medium at 48 h of incubation.

Bacterial Species	Strain Code	Growth Medium	Temperature (°C)	Medium Renewal (h)
*S.* Typhimurium	FMCC_B137	1/10 TSB ^1^	20	48
*L. monocytogenes*	AAL 20107	BHI ^2^ broth	37	48
*Y. enterocolitica*	DSM 4780	TSB	20	48

^1^ Trypticase Soya Broth; ^2^ Brain Heart Infusion.

## Data Availability

The data presented in this study are available upon reasonable request from the corresponding author.

## Supplementary Materials

The following supporting information can be downloaded at: https://www.mdpi.com/article/10.3390/molecules28031154/s1, Figure S1: Bioscreen C° Pro growth curves (A_6000 nm_) of *S*. Typhimurium, *L. monocytogenes*, and *Y. enterocolitica* strains during their 24-h growth in TSB at 37 °C in the presence of ten different *T. capitatus* EO concentrations (two-fold dilutions ranging from 0.50 to 0.001 % *v*/*v*). For each bacterial strain, the growth curves of the positive (PC; TSB inoculated with bacteria also containing 0.6% *v*/*v* EtOH) and the negative control (NC; sterile TSB) are also shown, together with the determined MIC value (no increase in broth’s absorbance with respect to the negative control during whole incubation). The bars of standard deviations of the planktonic means were omitted for clarity.

## Sample Availability

Samples of the compounds are not available from the authors.
