# Supplementary material for: Chemical Composition, Antioxidant, and Antibiofilm Properties of Essential Oil from Thymus capitatus Plants Organically Cultured on the Greek Island of Lemnos
Source: Molecules. 2023 Jan 24;28(3):1154. doi: 10.3390/molecules28031154 (PMC9919994; doi:10.3390/molecules28031154)
Supplement: Supplementary file 1 [file molecules-28-01154-s001.zip › molecules-2152915-supplementary.pdf]

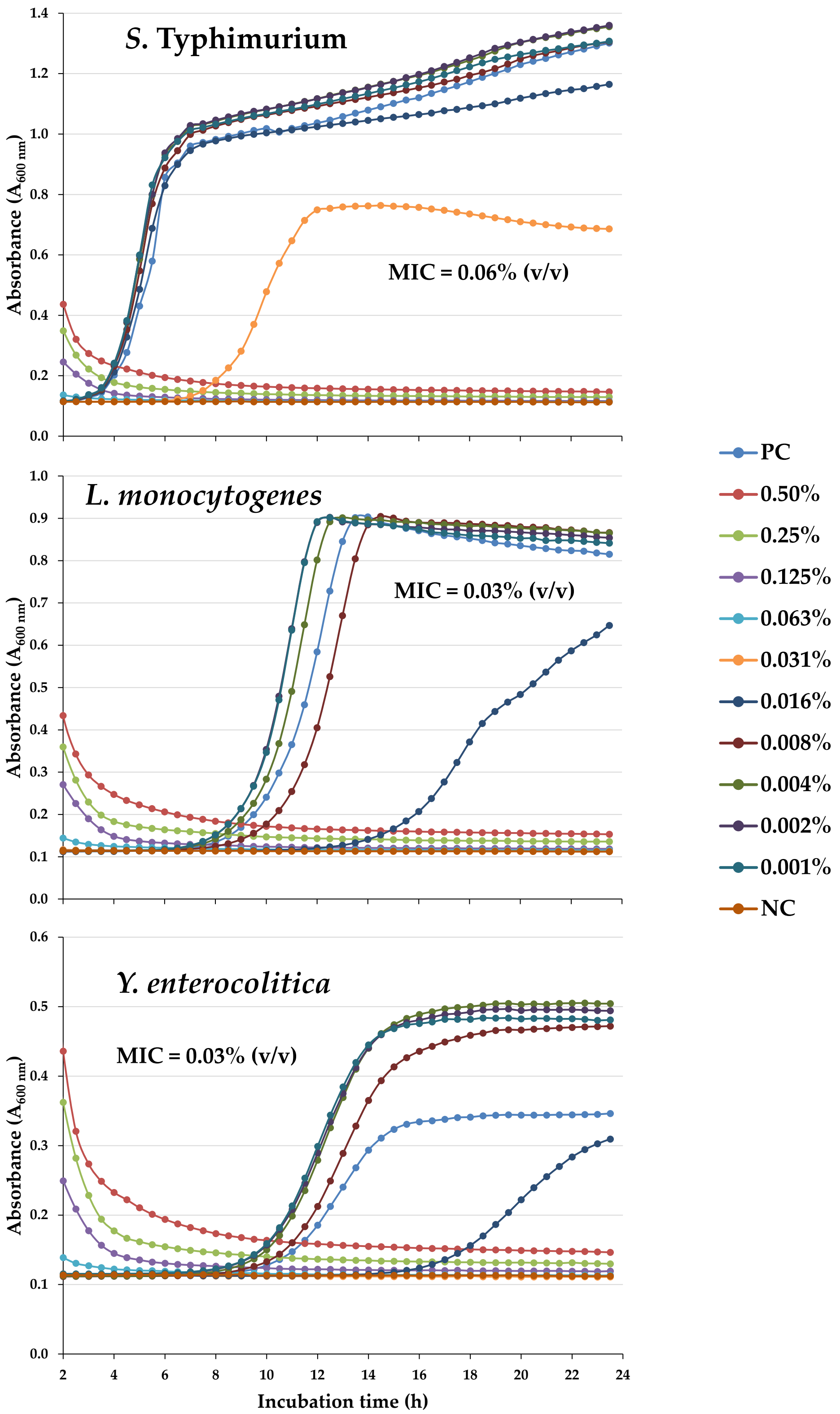

Figure S1. Bioscreen C° Pro growth curves ($A_{6000\ nm}$) of *S.* Typhimurium, *L. monocytogenes*, and *Y. enterocolitica* strains during their 24-h growth in TSB at 37 °C in the presence of ten different *T. capitatus* EO concentrations (two-fold dilutions ranging from 0.50 to 0.001 % v/v). For each bacterial strain, the growth curves of the positive (PC; TSB inoculated with bacteria also containing 0.6% v/v EtOH) and the negative control (NC; sterile TSB) are also shown, together with the determined MIC value (no increase in broth's absorbance with respect to the negative control during whole incubation). The bars of standard deviations of the planktonic means were omitted for clarity.
